# Suppression of death receptor 5 enhances cancer cell invasion and metastasis through activation of caspase-8/TRAF2-mediated signaling

**DOI:** 10.18632/oncotarget.5847

**Published:** 2015-10-15

**Authors:** You-Take Oh, Ping Yue, Dongsheng Wang, Jing-Shan Tong, Zhuo G. Chen, Fadlo R. Khuri, Shi-Yong Sun

**Affiliations:** ^1^ Department of Hematology and Medical Oncology, Emory University School of Medicine and Winship Cancer Institute, Atlanta, GA, USA; ^2^ Department of Pharmacology and Chemical Biology, University of Pittsburgh Cancer Institute and School of Medicine, Pittsburgh, PA, USA

**Keywords:** death receptor 5, invasion, metastasis, caspase-8, TRAF2

## Abstract

The role of death receptor 5 (DR5), a well-known cell surface pro-apoptotic protein, in the negative regulation of invasion and metastasis of human cancer cells and the underlying mechanisms are largely unknown and were hence the focus of this study. In this report, we have demonstrated that DR5 functions to suppress invasion and metastasis of human cancer cells, as evidenced by enhanced cancer cell invasion and metastasis upon genetic suppression of DR5 either by gene knockdown or knockout. When DR5 is suppressed, FADD and caspase-8 may recruit and stabilize TRAF2 to form a metastasis and invasion signaling complex, resulting in activation of ERK and JNK/AP-1 signaling that mediate the elevation and activation of matrix metalloproteinase-1 (MMP1) and eventual promotion of cancer invasion and metastasis. Our findings thus highlight a novel non-apoptotic function of DR5 as a suppressor of human cancer cell invasion and metastasis and suggest a basic working model elucidating the underlying biology.

## INTRODUCTION

Death receptor (DR5; also called TRAIL-R2 or Killer/DR5) is a death domain-containing transmembrane cell surface protein. Upon binding to its ligand, tumor necrosis factor-related apoptosis-inducing ligand (TRAIL), or induction of aggregation or clustering (e.g., by overexpression of DR5 or with an agonistic antibody), DR5 is activated and subsequently initiates apoptosis through interaction with the adaptor protein, Fas-associated death domain (FADD), which further recruits and activates caspase-8 [1, 2]. Hence, the primary function of DR5 is to induce apoptosis. Because of the selectivity of TRAIL towards cancer cells, there has been significant interest in developing agents targeting TRAIL or DR5 for cancer therapy, including recombinant protein, agonistic antibodies and small molecules [3, 4].

Although DR5 has been considered a potential cancer therapeutic target, its precise physiological or biological role in the regulation of human cancer development remains unclear [3, 5]. Mice deficient in mouse TRAIL death receptor (mDR; the sole mouse ortholog of human DR4 and DR5) show increased susceptibility to tumorigenesis, such as Myc-driven lymphoma and diethylnitrosamine-induced hepatocarcinogenesis [6]. However, the loss of *mDR* did not influence the incidence of lymphomas in p53-null mice or intestinal tumor development in adenomatous polyposis coli mutant mice (APCmin model) [7]. Some studies with human tissue specimens indicate that DR5 is overexpressed in several cancer types and significantly correlated with more aggressive tumor behavior and poor survival of cancer patients (e.g., with breast, lung or renal cell cancer) [8–10]. However other studies show that DR5 expression (e.g., in bladder or colorectal cancer) is associated with a less aggressive phenotype and better survival or longer postoperative recurrence-free rate [11, 12]. In some types of cancers (e.g., ovarian and cervical cancer), DR5 expression does not impact cancer patient survival [13, 14].

Metastasis is a hallmark stage of cancer development or progression, representing an inefficient process involving multistep events, in which only a small proportion of the many cells that migrate from the primary tumor successfully colonize distant sites [15]. Cancer-related deaths occur largely due to the development of uncontrolled metastases. Generally, metastatic cells must first detach from the primary tumor mass and be able to survive in an anchorage-independent manner. Subsequently, the surviving cells must navigate the lymphatic and circulatory channels while at the same time evading immune surveillance. Circulating tumor cells must possess the cellular machinery to invade distal organs, implant within local tissues, and initiate *de novo* tumor growth [15, 16].

It has been shown that mDR deficiency in mice enhances lymph node metastasis of skin carcinoma [17] and metastasis of lymphoma cells to liver and lung during c-myc-driven lymphomagenesis [6], suggesting that mDR may be critical for the negative regulation of tumor metastasis. In human melanoma tumor samples, a reduced DR5 expression was reported to be associated with metastatic lesions [18]. Our study with head and neck cancer specimens showed a significant reduction of DR5 expression in primary tumors with metastasis and their matching lymph node metastasis compared to primary tumors with no evidence of metastasis [19]. Interestingly, approximately 12% of inactivating mutations primarily in the death domain of DR5 were detected exclusively in breast cancer with lymph node metastasis, but not in breast cancer without metastasis [20]. Moreover, it has recently been shown that the DR5 agonistic antibody lexatumumab robustly suppresses lymph node or lung metastasis in an orthotopic model of triple-negative breast cancer [21]. These findings support the notion that DR5 may be associated with suppression of cancer metastasis. However, another study has suggested that oncogenic K-Ras and its effector, Raf1, can convert death receptors (e.g., Fas and DR5) into invasion-inducing receptors by suppressing the ROCK/LIM kinase pathway, and this is essential for K-Ras/Raf1-driven metastasis formation [22].

Therefore, it is unclear whether DR5 indeed plays a role in the regulation of cancer invasion and metastasis in humans. The current study aimed to determine the involvement of DR5 in the regulation of human cancer cell invasion and metastasis and to understand the underlying biology or mechanisms. Through genetic manipulation of DR5 expression in human cancer cells, we have shown that DR5 does indeed function as a suppressor of cancer invasion and metastasis, primarily via modulating caspase-8/TRAF2-mediated signaling.

## RESULTS

### Suppression of DR5 expression enhances the invasive capacities of cancer cells

**Figure 1:**
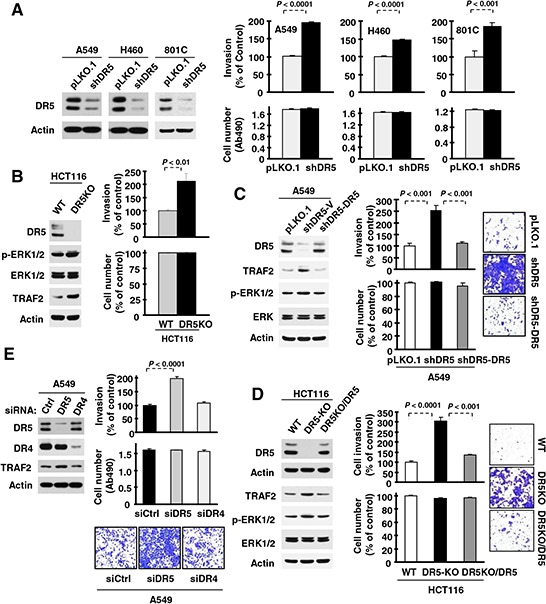
Suppression of DR5, but not DR4, expression increases cancer cell invasion **A–D.** The indicated cell lines, in which DR5 was stably knocked down (A), knocked out (B), knocked down and then re-expressed (C) or knocked out and then re-expressed (D) as evaluated with Western blotting were plated in Matrigel invasion chambers for cell invasion assay and in 96-well plates for cell number estimation with MTS assay after approximately 48 h incubation. **E.** A549 cells transiently transfected with control (Ctrl), DR5 or DR4 siRNA were plated in 12-well plates for evaluation of DR5 or DR4 expression by Western blotting, in the Matrigel invasion chambers for invasion assay and in 96-well plates for cell number estimation after approximately 48 h incubation. The data are means ± SDs of triplicate (invasion) or quadruplicate (cell growth) determinations. WT, wild-type.

We first studied the impact of gene silencing-mediated DR5 suppression on cancer cell invasion. Knockdown of DR5 expression with short-hairpin RNA (shRNA) did not affect the growth of several tested human cancer cell lines including A549, H460 and 801C, but significantly enhanced their invasive abilities (Fig. 1A). Similar results were also generated with small interfering RNA (siRNA) specific for DR5 (Supplemental Fig. S1). In agreement, the DR5-knockout (KO) HCT116 cell line exhibited significantly higher invasive capacity than its isogenic parental cell line carrying wild-type (WT) DR5 (Fig. 1B). When DR5 was re-expressed in A549-shDR5 cells or in HCT116-DR5KO cells, the enhanced invasive phenotype observed was abolished (Figs. 1C and 1D). These results together robustly indicate that significantly increased invasion is indeed a specific consequence of DR5 suppression. DR4 is a functional sibling of DR5 with almost identical function in mediating TRAIL-induced apoptosis. Interestingly we found that knockdown of DR5 expression, but not DR4 expression, increased cancer cell invasion (Fig. 1E). Hence we conclude that DR5 has a suppressive role in the regulation of cancer cell invasion.

### DR5 suppression increases *in vivo* lung metastasis

**Figure 2:**
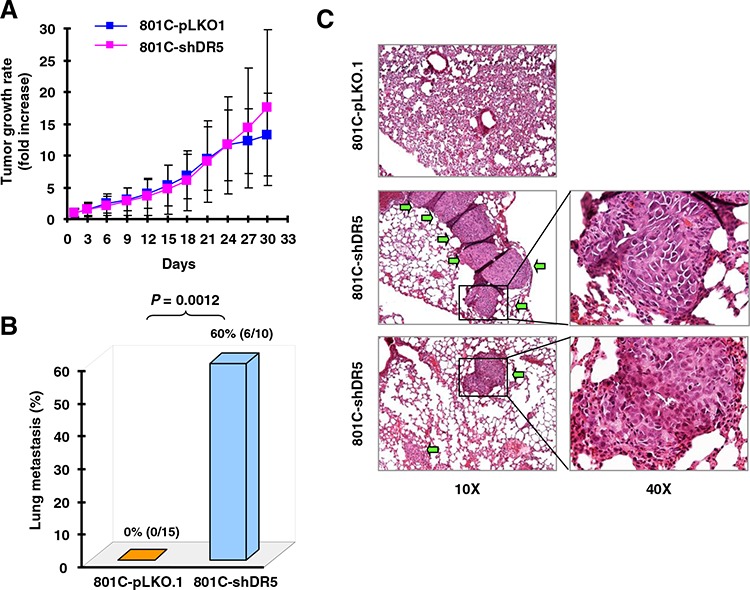
Knockdown of DR5 increases cancer cell metastasis *in vivo* Both 801C-pLKO.1 and 801C-shDR5 cells were injected subcutaneously into nude mice. After approximately one month, the mice began to be sacrificed to collect lungs for evaluation of human cancer cell metastasis with H&E staining of lung slices. Primary tumor growth rates **(A)**, lung metastasis incidences **(B)**, and representative pictures of lung metastasis **(C)** are presented. The areas indicated by arrows are metastasized human cancer nodules.

Next, we determined whether DR5 suppression also increases cancer metastasis *in vivo*. To this end, we used the PLA-801C lung cancer cell line, which has low metastatic potential and exhibited enhanced invasion upon DR5 knockdown with either siRNA or shRNA (Figs. 1 and S1). We injected both 801C-pLKO.1 control cells and 801C-shDR5 cells subcutaneously into nude mice and then evaluated tumor growth rates and lung metastasis. Both xenografts had comparable growth rates (Fig. 2A). However, we detected lung metastasis in 60% (6/10) of mice with shDR5 xenografts, but not in any mice (0%; 0/15) carrying pLKO.1 xenografts (Figs. 2B and 2C). Hence, DR5 knockdown significantly enhanced lung metastasis of cancer cells *in vivo* (*P* = 0.0012) without altering primary tumor growth. This result provides strong *in vivo* evidence in support of the role of DR5 in suppressing cancer metastasis.

### DR5 suppression increases TRAF2 levels and activates ERK and JNK/AP-1 signaling accompanied with MMP1 elevation

**Figure 3:**
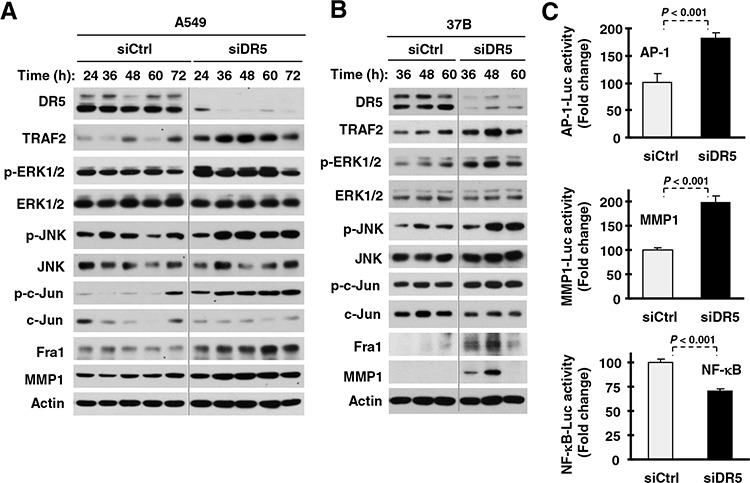
DR5 knockdown activates ERK and JNK/AP-1 signaling, increases TRAF2 levels and enhances MMP expression **A** and **B.** The indicated cell lines were transfected with control (Ctrl) or DR5 siRNA and then harvested after different times as indicated for preparation of whole-cell protein lysates and subsequent Western blotting. **C.** A549 cells were co-transfected with AP-1, MMP1 or NF-κB luciferase reporter construct and control (Ctrl) or DR5 siRNA. After 42 h, the cells were lysed for luciferase activity assay. Each column represents a mean ± SD of triplicate determinations.

To understand the biology underlying the DR5-dependent regulation of cancer cell invasion, we examined how DR5 suppression affects signaling pathways and proteins involved in the regulation of cell invasion. We found that the levels of matrix metalloproteinase-1 (MMP1), a well-known protein implicated in mediating cell invasion [23], were higher in DR5 siRNA-transfected cells than in control siRNA-transfected cells. Since the expression of MMP1 is regulated by JNK/AP1 and ERK signaling [23], we then examined alterations in these signaling pathways. The levels of *p*-ERK1/2, *p*-JNK, *p*-c-Jun and Fra-1 were also elevated in DR5 siRNA-transfected cells compared with those in control siRNA-transfected cells (Figs. 3A and 3B). These data suggest that knockdown of DR5 activates the ERK1/2 and JNK/AP-1 signaling pathways and elevates MMP1 levels. In agreement, knockdown of DR5 increased AP-1 and MMP1 luciferase activity (Fig. 3C), supporting the notion that DR5 knockdown increases AP-1 transcriptional activity. Interestingly, the levels of TRAF2, a protein involved in mediating TRAIL/DR survival signaling [24] and in positively regulating NF-κB activity and cell invasion [25], were also elevated in DR5 siRNA-transfected cells (Figs. 3A and 3B). However, we did not see an increase in NF-κB activity when DR5 was silenced (Fig. 3C).

Consistently, we detected increased levels of TRAF2 and *p*-ERK1/2 in DR5-KO HCT116 cells in comparison with their parental cells (Fig. 1B). Re-expression of DR5 in A549-shDR5 or HCT116-DR5KO cells prevented elevation of both TRAF2 and *p*-ERK1/2 induced by DR5 knockdown (Fig. 1C) or knockout (Fig. 1D). Therefore, these data further support the notion that DR5 suppression activates ERK signaling and elevates TRAF2 levels.

### Activation of ERK and JNK/AP-1 signaling contributes to DR5 suppression-induced invasion of cancer cells

**Figure 4:**
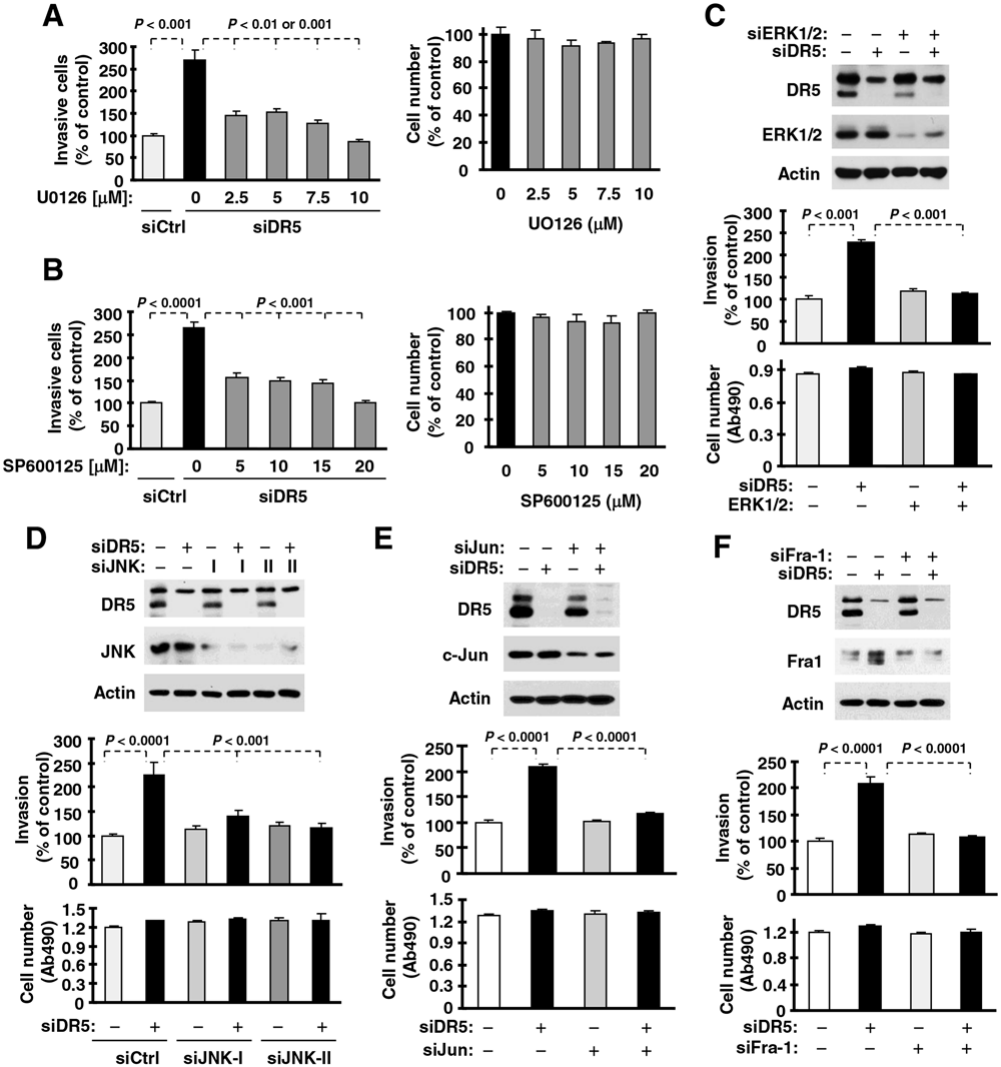
ERK (A and C), JNK (B and D), c-Jun (E) and Fra-1 (F) mediate DR5 knockdown-induced cell invasion **A** and **B.** A549 cells transfected with control (Ctrl) or DR5 siRNA were plated in Matrigel invasion chambers for approximately 12 h and then exposed to the given concentrations of U0126 (A) or SP600125 (B) in the bottom wells for an additional 36 h. The invasive cells were stained and measured. In addition, cell growth in 96-well plates was measured with the MTS assay after exposure to U0126 or SP600125 for about 48 h. **C–F.** A549 cells were co-transfected with the indicated siRNAs alone or in combinations and then seeded in 12-well plates for Western blotting to confirm knockdown efficiencies, in Matrigel invasion chambers for cell invasion assays, and in 96-well plates for cell growth measurements after approximately 48 h incubation. The data are means ± SDs of triplicate (invasion) or quadruplicate (cell growth) determinations.

To determine whether the observed activation of ERK and JNK/AP-1 signaling is involved in the increased invasion induced by DR5 knockdown, we used both chemical and genetic approaches. In the presence of either the MEK inhibitor U0126 or the JNK inhibitor SP600125, the ability of DR5 knockdown to increase cell invasion was inhibited or abrogated (Figs. 4A and 4B). Under the tested conditions, these inhibitors minimally affected cell growth. In agreement, inhibition of ERK or JNK by gene silencing with the more specific siRNA approach also blocked DR5-induced promotion of cell invasion (Figs. 4C and 4D). Moreover knockdown of c-Jun or Fra-1, both of which are key components of the AP1 complex, inhibited promotion of cell invasion induced by DR5 knockdown (Figs. 4E and 4F). Therefore, it is clear that the activation of both ERK and JNK/AP1 is required for promotion of cell invasion induced by DR5 knockdown.

### ERK- and JNK/AP1-dependent MMP1 elevation mediates DR5 knockdown-induced cancer cell invasion

**Figure 5:**
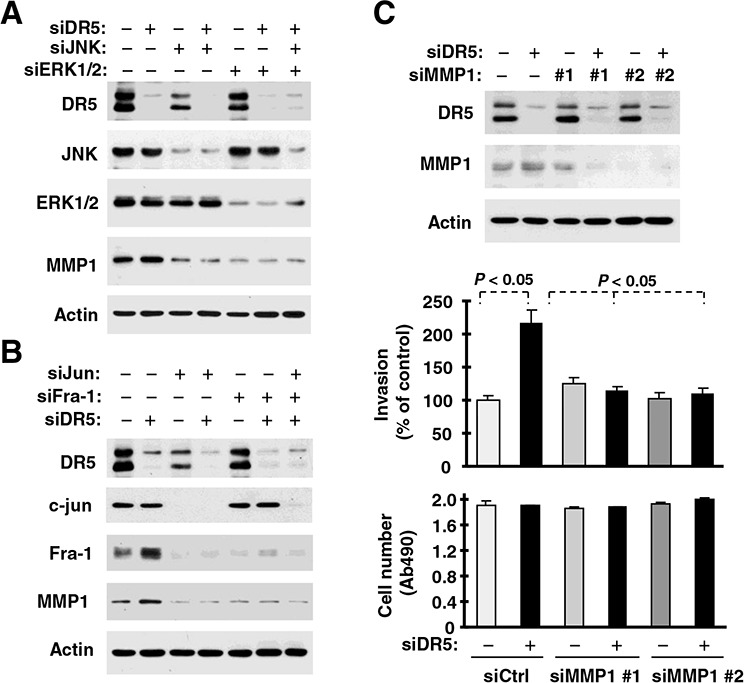
DR5 knockdown leads to ERK-, JNK-, c-Jun- and Fra-1-dependent MMP1 elevation (A and B) and enhanced cell invasion (C) **A.** and **B.** A549 cells were transfected with the indicated siRNAs alone or in combinations and after 48 h, were harvested for preparation of whole-cell protein lysates and subsequent Western blot analysis. **C.** A549 cells transfected with the indicated siRNAs were seeded in 12-well plates for Western blotting, in Matrigel invasion chambers for cell invasion assay and in 96-well plates for cell growth measurements after approximately 48 h incubation. The data are means ± SDs of duplicate (invasion) or quadruplicate (cell growth) determinations.

Since the expression of MMP1 is known to be regulated by JNK/AP1 and ERK signaling [23], we next asked whether activation of JNK/AP-1 or ERK mediates MMP1 upregulation induced by DR5 knockdown in our cell systems. Inhibition of JNK, ERK or both by knocking down their expression singly and in combination not only decreased basal levels of MMP1, but also blocked DR5 knockdown-induced MMP1 elevation (Fig. 5A). Consistently, inhibition of AP1 by knockdown of c-Jun, Fra-1 or both reduced basal levels of MMP1 and abolished DR5 knockdown-induced MMP1 upregulation (Fig. 5B). Hence, these data lead convincingly to the conclusion that the activation of JNK/AP-1 and ERK signaling mediates MMP1 upregulation induced by DR5 knockdown. Following these studies, we further determined whether MMP1 is indeed critical for mediating cell invasion induced by DR5 knockdown. As shown in Fig. 5C, DR5 knockdown promoted cell invasion in control siRNA-transfected cells, but not in cells transfected with MMP1 siRNAs, indicating that MMP1 elevation or activation is indeed critical for DR5 knockdown-induced promotion of cell invasion.

### Caspase-8, FADD and TRAF2 are all required to mediate promotion of cell invasion induced by DR5 knockdown

**Figure 6:**
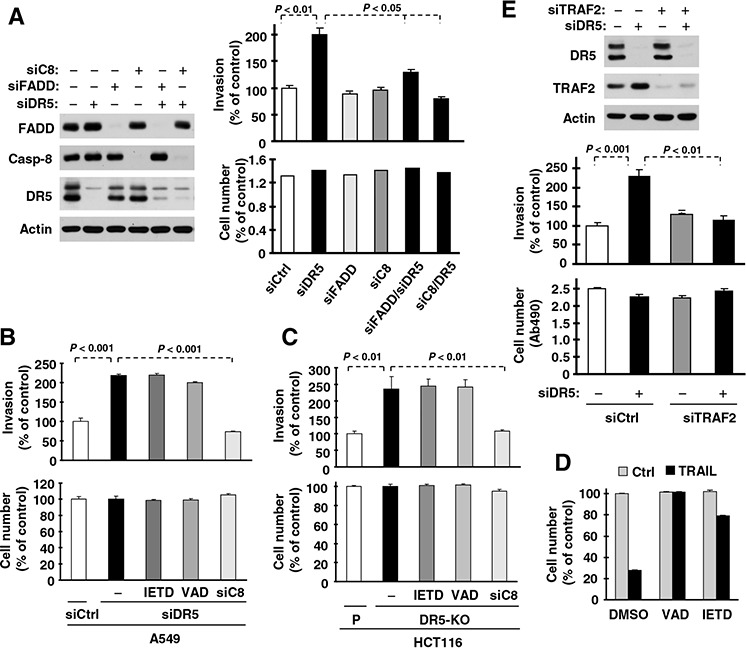
DR5 suppression-induced promotion of cell invasion requires FADD, caspase-8 (A) and TRAF2 (E), but not caspase-8 activity (B–D) **A** and **E.** A549 cells transfected with the indicated siRNAs alone or in combinations were seeded in 12-well plates for Western blotting to detect the given proteins, in Matrigel invasion chambers for cell invasion assays and in 96-well plates for cell growth measurements after approximately 48 h incubation. The data are means ± SDs of triplicate (A) or duplicate (E) determinations (invasion) or quadruplicate determinations (cell growth). **B** and **C**, A549 (B) and HCT116 DR5-KO (C) cells transfected with control (Ctrl) or indicated siRNA were plated in Matrigel invasion chambers for approximately 12 h and then exposed to the given caspase inhibitors (20 μM) in the bottom wells for an additional 36 h. The invasive cells were stained and measured. In addition, the cells were also plated in 96-well plates and received the same treatments with the indicated caspase inhibitors for 48 h for measurement of cell growth with the MTS assay. The data are means ± SDs of triplicate (invasion) or quadruplicate (cell growth) determinations. **D**, HCT116 cells were seeded in 96-well plates and treated with 50 ng/ml TRAIL alone or in combination with 20 μM Z-VAD or Z-IETD. After 4 h, the cell viability was measured with the MTS assay. The data are means ± SDs of quadruplicate determinations.

FADD and caspase-8 are known to be key proteins in mediating DR5 apoptotic and survival signaling [24]. To further understand the biology underlying DR5 suppression-induced promotion of cell invasion, we examined whether FADD and caspase-8 are involved in this process. Through a genetic siRNA approach, we found that knockdown of either FADD or caspase-8 attenuated or blocked promotion of cell invasion induced by DR5 knockdown (Fig. 6A). Therefore, both FADD and caspase-8 are required to mediate DR5 knockdown-induced promotion of cell invasion. Moreover we determined whether caspase-8 activity is required for mediating enhanced cell invasion induced by DR5 suppression. We found that caspase-8 knockdown, but not Z-VAD-FMK (a pan-caspase inhibitor) or Z-IETD-FMK (a caspase-8 inhibitor), abrogated cell invasion enhanced by DR5 knockdown (Fig. 6B) or by DR5 knockout (Fig. 6C). At the tested concentrations, both Z-VAD-FMK and Z-IETD-FMK effectively blocked TRAIL-induced cell death (Fig. 6D), indicating an effective inhibition of caspase-8 activity. Hence caspase-8 activity is clearly not required for mediating enhanced invasion by DR5 suppression.

TRAF2 is another important component of the DR5-related complex mediating DR5 survival signaling [24]. As demonstrated above, TRAF2 levels are elevated in cells in which DR5 expression is suppressed. Hence we further determined the role of TRAF2 elevation in DR5 knockdown-induced cell invasion. As shown in Fig. 6E, we detected increased cell invasion in DR5 siRNA-transfected cells, but not in cells co-transfected with DR5 and TRAF2 siRNAs, indicating that TRAF2 elevation is also required for DR5 suppression-induced promotion of cell invasion.

### DR5 suppression results in caspase-8-dependent upregulation of TRAF2, activation of JNK/AP-1 and ERK1 signaling and elevation of MMP1

**Figure 7:**
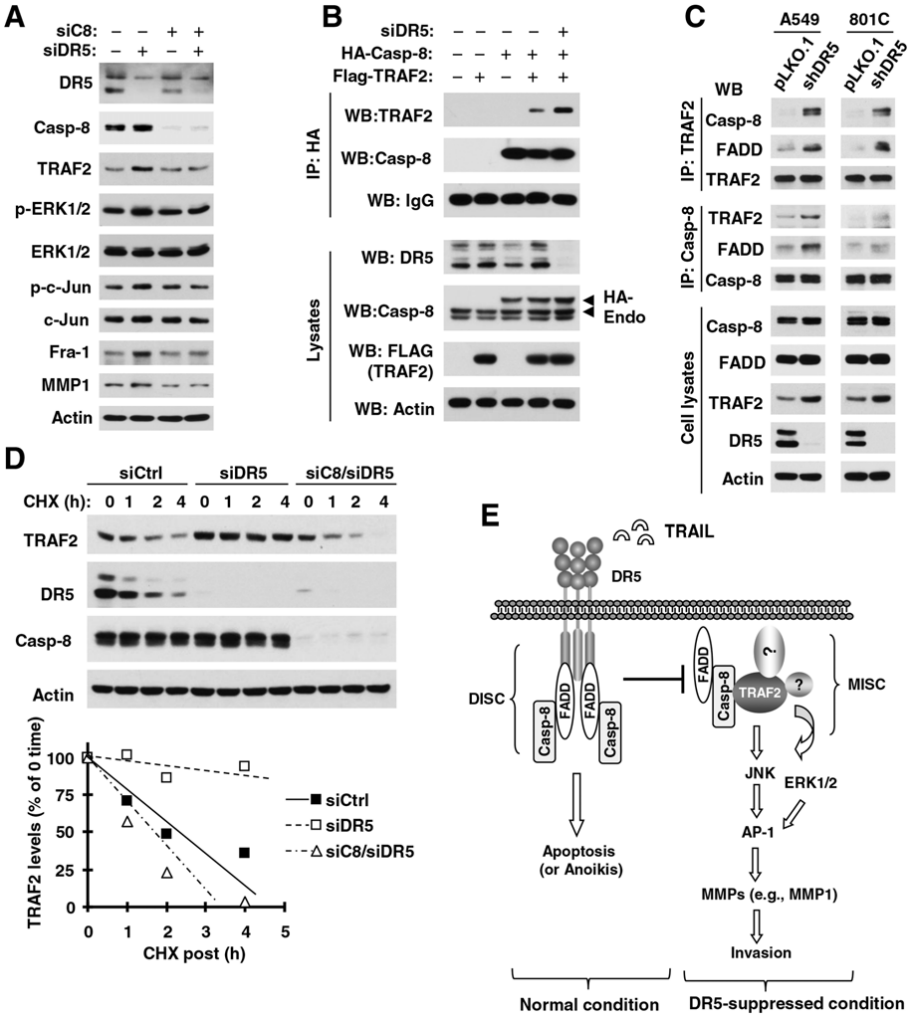
DR5 knockdown induces caspase-8-dependent elevation of TRAF2 and MMP1 (A), activation of ERK and JNK/AP-1 signaling (A) and stabilization of TRAF2 (D), and promotes caspase-8 interaction with TRAF2 (B and C); these events constitute a basic working model for how DR5 suppression leads to enhanced cancer cell invasion and metastasis (E) **A.** A549 cells were transfected with the indicated siRNAs alone or in combinations and after approximately 48 h were harvested for preparation of whole-cell protein lysates and subsequent Western blotting to detect the given proteins. **B.** HEK293T cells were co-transfected with Flag-TRAF2, HA-Caspase-8, and DR5 siRNA. After 33 h, whole-cell protein lysates were prepared from these cells and subjected to IP and subsequent Western blotting (WB) for the indicated proteins. Endo, endogenous. **C.** Whole-cell protein lysates were prepared from the indicated cell lines and then subjected to IP with the given antibodies and subsequent Western blotting (WB) for different proteins as indicated. **D.** A549 cells were transfected with control (Ctrl), DR5 or caspase-8 plus DR5 siRNA. After 36 h, the cells were treated with CHX (20 μg/ml) and then harvested at the indicated time points for preparation of whole-cell protein lysates. The indicated proteins were detected with Western blotting. Protein levels were quantified with NIH Image J software (Bethesda, MA) and normalized to actin. The results were plotted as the relative TRAF2 levels compared to those at time 0 of CHX treatment. **E.** A working model for DR5-mediated suppression of cancer cell invasion is shown. The primary function of DR5 is to mediate apoptosis upon activation through formation of the DISC; this will restrict the formation of the MISC, and subsequently suppress cell invasion. When DR5 is suppressed, available FADD and caspase-8 may recruit and stabilize TRAF2, resulting in the activation of ERK and JNK signaling and subsequent AP1-dependent expression and activation of MMPs (e.g., MMP1) and final promotion of invasion and metastasis of cancer cells.

Given the critical role of caspase-8 in mediating DR5 signaling, we then asked whether caspase-8 is involved in the elevation of TRAF2 and MMP1 and activation of JNK/AP-1 and ERK1 signaling induced by DR5 suppression. To this end, we compared the effects of knockdown of DR5 and caspase-8 alone and in combination on these events. As indicated in Fig. 7A, we detected increased levels of TRAF2, *p*-ERK1/2, *p*-c-Jun, Fra-1 and MMP1 in cells transfected with DR5 siRNA, but not in cells transfected with the combination of DR5 and caspase-8 siRNAs. Thus, caspase-8 is critical for mediating elevation of TRAF2 and MMP1 and activation of JNK/AP-1 and ERK signaling induced by DR5 suppression.

### DR5 suppression enhances caspase-8 and TRAF2 interaction and caspase-8-dependent TRAF2 stability

Following these findings, we were interested in determining how DR5 suppression increases caspase-8-dependent TRAF2 elevation. We first determined whether caspase-8 interacts with TRAF2 and whether DR5 knockdown impacts this interaction. Through an immunoprecipitation (IP) experiment with anti-HA antibody, we detected both HA-tagged caspase-8 and TRAF2 in cells transfected with both ectopic caspase-8 and TRAF2, suggesting a potential interaction between these proteins. Moreover we detected much higher amounts of TRAF2 in cells transfected with DR5 siRNA than in those without DR5 siRNA transfection (Fig. 7B), suggesting that DR5 knockdown enhances caspase-8 and TRAF2 interaction. We also conducted similar IP experiments with either anti-caspase-8 or anti-TRAF2 antibody to validate this finding by detecting their endogenous interaction. In both A549 and 801C cell lines, we detected higher amounts of both TRAF2 and FADD in shDR5 cells than in pLKO.1 control cells when caspase-8 was pulled down. Complementarily, we detected higher amounts of both caspase-8 and FADD in shDR5 cells than in pLKO.1 control cells when TRAF2 was immunoprecipitated (Fig. 7C). These results clearly suggest that DR5 knockdown increases the interaction or association of caspase-8 not only with TRAF2, but also with FADD.

Furthermore, we compared the stability of TRAF2 between cells transfected with control and DR5 siRNAs and found that the TRAF2 degradation rate was much slower in DR5 siRNA-transfected cells than in control siRNA-transfected cells (Fig. 7D). This finding indicates that DR5 knockdown stabilizes TRAF2 protein; this may account for the elevated level of TRAF2 caused by DR5 suppression. When caspase-8 was co-silenced with DR5 in cells, the TRAF2 degradation rate was enhanced and was comparable with that in cells transfected with control siRNA only (Fig. 7D). Thus it is clear that DR5 knockdown slows TRAF2 degradation, resulting in TRAF2 stabilization or elevation in a caspase-8-dependent manner.

## DISCUSSION

DR5 is well known to mediate apoptosis upon ligation with its ligand or induction of its clustering or aggregation (e.g., with an agonistic antibody or overexpression). In this study, we showed that inhibition of DR5 by knockdown or knockout increased invasion of human cancer cells. Strikingly, knockdown of DR5 expression significantly increased lung metastasis of cancer cells in a nude mouse subcutaneous lung cancer xenograft model (Figures 1 and 2). These data strongly suggest that DR5 plays a negative role in the regulation of human cancer cell invasion and metastasis in addition to its apoptosis-inducing function, hence warranting further study in this direction. This finding is in agreement with previous reports that mDR knockout in mice increases metastasis of skin carcinoma cells to lymph node metastasis during DMBA/TPA-induced skin carcinogenesis [17] and metastasis of lymphoma cells to liver and lung during c-myc-driven lymphomagenesis [6].

While we attempted to publish our results, a recent pulication reports that mDR and human DR5 promotes K-Ras-driven cancer progression, invasion and metastasis [26], although these results are contradictory to their previous findings using a H-Ras-driven skin carcinogenesisi model [17]. The cell lines used in our study including A549, H460 and HCT116 all have mutant K-Ras. Under our experimental condictions, we did not see reduction of invasison in these cell lines upon DR5 suppression. Nontheless, our *in vitro* and *in vivo* results consistently show that DR5 suppression enhances cancer cell invasion and metastasis. Further investigations for clarification of the discrepancy may be needed in the future.

It has been suggested that, in addition to the primary death-inducing signaling complex (DISC) formation and induction of apoptosis, engagement of DR5 can induce the formation of a secondary signaling complex, which mainly contains the core DISC components, FADD and caspase-8, as well as RIP1 and TRAF2, leading to activation of additional signaling cascades, including the NF-κB, ERK, JNK and p38 signaling pathways [3, 27]. Our findings show that DR5 suppression actually activates ERK and JNK/AP-1 signaling, contributing to the promotion of cancer cell invasion induced by DR5 knockdown. Thus, a different underlying mechanism accounting for activation of ERK and JNK/AP-1 signaling may exist.

MMP1 has long been implicated in mediating cell invasion and its expression is regulated by JNK/AP1 and ERK signaling [23]. In this study, DR5 knockdown increased MMP1 transcriptional activity and elevated its protein levels. Moreover, suppression of MMP1 by knocking down its expression abrogated DR5 knockdown-induced promotion of cell invasion (Fig. 5). Collectively, we believe that MMP1 activation is critical for mediating increased cell invasion induced by DR5 knockdown. Additionally, we showed that inhibition of JNK, ERK or AP-1 (c-Jun or Fra1) with siRNA-mediated gene silencing blocked MMP1 elevation induced by DR5 knockdown (Fig. 5), demonstrating ERK- and JNK/AP-1-dependent upregulation of MMP1. Therefore, we conclude that inhibition of DR5 promotes cell invasion through ERK1- and JNK/AP-1-mediated MMP1 elevation. In this study, we did not explore the alteration of other MMPs in cells transfected with DR5 siRNA and therefore cannot rule out the possible involvement of other MMPs in mediating DR5 suppression-induced enhancement of cell invasion.

In our study, TRAF2 levels were increased in cells in which DR5 was knocked down (Fig. 3). Furthermore, while knockdown of DR5 alone facilitated cell invasion, knockdown of both DR5 and TRAF2 failed to promote cell invasion (Fig. 6E). Although DR5 knockdown did not alter the levels of FADD and caspase-8, siRNA-mediated depletion of FADD or caspase-8 abrogated the ability of DR5 knockdown to promote cell invasion (Fig. 6A). These results clearly demonstrate that these proteins are all required for DR5-mediated negative regulation of cell invasion. In support of this notion, our data further showed that depletion of caspase-8 abrogated elevation of TRAF2 and MMP1 and activation of ERK and JNK/AP-1 signaling induced by DR5 knockdown (Fig. 7A).

Moreover, we have shown that DR5 knockdown increases TRAF2 levels by stabilization of TRAF2, as evidenced by the slowed degradation of TRAF2 upon DR5 knockdown (Fig. 7D). Co-knockdown of caspase-8 and DR5 reversed the effects of DR5 knockdown on TRAF2 elevation (Fig. 7A) and stabilization (Fig. 7D), clearly indicating that DR5 suppression causes caspase-8-dependent stabilization and elevation of TRAF2. In addition, we detected an interaction between caspase-8 and TRAF2; this interaction was further enhanced when DR5 was knocked down (Figs. 7B and 7C). In agreement, we found that knockdown of caspase-8, but not inhibition of its activity (e.g., by caspase inhibitors), was able to abrogate the ability of DR5 suppression to increase cell invasion (Figs. 6B and 6C), indicating the importance of caspase-8 protein, rather than its enzymatic activity, in enhancing cell invasion induced by DR5 suppression. Based on these data, we suggest that DR5 suppression enhances caspase-8 interaction with TRAF2, resulting in stabilization of TRAF2.

Taking all our data together, we propose a working model as follows: the activation of DR5 normally favors formation of the DISC, resulting in induction of apoptosis or anoikis as well as other potential biological consequences; this will not only lead to direct killing of detached cancer cells (e.g., via anoikis or TRAIL/DR5-mediated immunosurveillance), but also restrict the formation of another complex, the metastasis and invasion signaling complex (MISC), eventually resulting in suppression of cancer cell invasion and metastasis. When DR5 is inhibited (e.g., by mutation, deficiency or reduced expression), cancer cells will be resistant to anoikis or immunosurveillance. Available FADD and caspase-8 may recruit and stabilize TRAF2 (and perhaps other unknown proteins), resulting in the activation of ERK and JNK signaling and subsequent AP-1-dependent expression and activation of MMPs (e.g., MMP1) and final promotion of invasion and metastasis of cancer cells (Fig. 7E). Further investigation in this direction is warranted.

Although DR5 and DR4 share redundant functions and mechanisms in mediating TRAIL-induced apoptosis, our study shows that DR4 does not have a similar role to DR5 in the negative regulation of cell invasion, since modulation of DR4 expression did not alter cell invasive capacity (Figs. 1E) or TRAF2 levels (Fig. 1E). Coincidently, a recent preclinical study showed that the DR5 agonistic antibody lexatumumab inhibited lymph node and lung metastasis more robustly than the DR4 agonistic antibody mapatumumab in an orthotopic model of triple-negative breast cancer [21]. Thus, it appears that DR5 and DR4 have distinct biological functions (e.g., regulation of cell invasion) while possessing some common functions (e.g., mediation of TRAIL-induced apoptosis). The molecular mechanisms underlying these distinct versus shared functions have yet to be elucidated, and thus further study in this direction is also warranted.

Mutations in DR5 have been identified in various human tumors [20, 28–31]. Most mutations identified so far are located in and affect the intracellular death domain of the receptor, a region essential for binding to FADD. For example, mutations in the death domain were detected in 10% of non-small cell lung cancers [28] and in 12% of breast cancers exclusively from patients with lymph node metastasis [20]. These tumor-derived mutations very often result in DR5 losing its ability to form a functional DISC and to induce apoptosis [32, 33]. They can also function as dominant-negative mutants to inhibit death signaling [32]. Therefore, this is a relevant example of DR5 suppression under a physiological or cancer-related condition. The fact that approximately 12% of DR5 mutations were detected exclusively in metastatic breast cancers [20] strongly supports our finding that DR5 suppression may have a critical role in promoting metastasis of human cancer.

## MATERIALS AND METHODS

### Reagents

The MEK inhibitor U0126 was purchased from LC Laboratories (Woburn, MA). The JNK inhibitor SP600125 was purchased from Enzo Life Sciences, Inc./Biomol (Farmingdale, NY). Rabbit polyclonal anti-DR5 antibody was purchased from ProSci Inc. (Poway, CA). Mouse monoclonal anti-DR4 antibody (B-N28) was purchased from Diaclone (Stamford, CT). Protein-A/G plus-agarose, rabbit polyclonal anti-TRAF2 (sc-7187) and Fra-1 antibodies were purchased from Santa Cruz Biotechnology, Inc (Santa Cruz, CA). Rabbit polyclonal antibody against HA tag was purchased from Abgent (San Diego, CA). Mouse monoclonal anti-MMP1 antibody was purchased from NeoMarkers, Inc. (Union City, CA). Antibodies against actin and Flag-tag and anti-Flag M2 affinity gel were purchased from Sigma-Aldrich (St. Louis, MO). Mouse monoclonal anti-TRAF2 (#558890) and anti-FADD (#556402) antibodies were purchased from BD Biosciences (San Jose, CA). Mouse monoclonal anti-caspase-8 (#9746), rabbit monoclonal anti-caspase-8 (#4790), rabbit polyclonal ant-FADD (#2782) and all other antibodies were all purchased from Cell Signaling Technology, Inc. (Beverly, MA). Anti-HA matrix agarose was purchased from Thermo Scientific/Pierce (Rockford, IL).

### Cell lines and cell culture

The human lung cancer cell lines A549, H460 and PLA-801C were described previously [34]. 37B and M4e head and neck cancer cell lines and HEK293T cells were provided by Drs. ZG Chen and K Ye (Emory University, Atlanta, GA), respectively. HCT116 and its isogenic DR5-KO cell lines were generously provided by Dr. L Zhang (University of Pittsburgh Cancer Institute, Pittsburgh, PA). Except for A549, H460 and PLA-801C cells, which were authenticated by analyzing short tandem repeat DNA profile, other cell lines have not been authenticated. These cell lines were cultured in RPMI 1640 or DMEM/F12 containing 5% fetal bovine serum at 37°C in a humidified atmosphere of 5% CO_2_ and 95% air.

### Western blot analysis

Whole-cell protein lysates were prepared and analyzed by Western blotting as described previously [35].

### Expression constructs and transfection

HA-tagged caspase-8 expression construct [36] was provided by Dr. K Vuori (Burnham Institute for Medical Research, La Jolla, CA). TRAF2 expression construct [37] was provided by Dr. H Habelhah (University of Iowa, Iowa City, IA). Generally, cells were transfected with the given plasmids using Lipofectamine™ 2000 (Invitrogen) as instructed by the manufacturer's protocol. Lentiviral DR5 expression construct was made by re-cloning DR5 cDNA in pCEP4 plasmid, which was originally obtained from Dr. WS El-Deiry (Fox Chase Cancer Center, Philadelphia, PA), into pLenti-Bi-cistronic vector (ABM Inc; BC, Canada) using PCR.

### Gene silencing using siRNA or shRNA

Gene silencing was achieved by either transfecting siRNA using HiPerFect transfection reagent (Qiagen, Valencia, CA) following the manufacturer's instructions or infecting cells with lentiviruses harboring a given shRNA. Control (i.e., non-silencing) and DR5-specific siRNAs were described previously [35]. DR5 shRNA in pLKO.1 (TRCN0000005929) targeting the 3′-untranslated region was purchased from Open Biosystems (Huntsville, AL). ERK1/2 (#6560), JNK (#6234 including #I and #II) and c-Jun (#6205) siRNAs were purchased from Cell Signaling Technology, Inc. TRAF2 (sc-29509), Fra-1 (sc-35405) and caspase-8 (sc-29930) siRNAs were purchased from Santa Cruz Biotechnology, Inc. MMP1 siRNAs that target the sequences of 5′-ACACAAGAGCAAGATGTGGAC-3′ (#1) and 5′-AAGTTGATGCAGTTTTCATGA-3′ (#2) were synthesized by Qiagen (Valencia CA). FADD siRNA was described previously [38]. Gene silencing effects were evaluated by Western blot analysis as described above.

### Re-expression of DR5 in shDR5 stable cell line and DR5-KO cell line

Both A549-shDR5 and HCT116-DR5KO cells were infected with lentiviruses, which were generated by transfecting pLenti-Bi-cistronic vector without or with DR5 cDNA together with packaging plasmids (ABM Inc) into 293T cells as instructed by the manufacturer, and followed by two-week selection with puromycin (2 μg/ml). The surviving cells were pooled and DR5 re-expression was confirmed with Western blotting.

### Reporter plasmids, transient transfection, and luciferase activity assay

The AP-1 (pAP1-luc) and NF-κB (pNF-κB-luc) luciferase reporter constructs were described previously [39, 40]. The reporter construct containing a −517/+63 bp 5′-flanking region of the MMP1 gene [41] was generously provided by Dr. AD Sharrocks (University of Manchester, Manchester, UK). The plasmid transfection and luciferase assays were the same as described previously [40].

### IP

The cells were lysed in RIPA buffer with protease and phosphatase inhibitors. The cell lysates were then incubated with anti-Flag M2 or anti-HA agarose at 4°C overnight according to the manufacturer's instruction (for tagged proteins) or with the anti-TRAF2 (sc-7187; Santa Cruz Biotechnology, Inc) or anti-caspase-8 (#9746; Cell Signaling Technology, Inc) antibody overnight followed by incubation with protein-A/G Plus-agarose for 8 h at 4°C. The beads were then washed four times (5 min each) with the same buffer used for cell lysis and boiled in 2 × SDS sample buffer for 5 min. Samples were then analyzed by SDS-PAGE followed by Western blotting.

### Cell invasion and growth assays

The *in vitro* cell invasion assay was carried out in BD BioCoat Matrigel invasion chambers (Becton Dickinson). Cancer cells in log-phase growth were detached by trypsin-EDTA (Mediatech) and re-suspended in growth media with 1% FBS. Cells (5 × 10^4^) transfected with the given siRNAs were seeded in the upper chambers, whereas the lower chambers contained 12% FBS medium. Following 36 h incubation at 37°C in a humidified 5% CO^2^ atmosphere, the non-invading cells in the upper chamber and on the Matrigel were mechanically removed with a cotton swab. The cells adherent to the lower surface of the membrane were fixed with 4% formaldehyde and stained with 0.2% crystal violet for 15 min. The cells that had migrated to the lower surface of the filter membrane were solubilized with 1% SDS. Absorbance was measured with a microplate reader at 570 nm. For cell proliferation analysis, transfected cells were plated onto 96-well plates at a density of 1 × 10^4^ per well with medium containing 5% FBS. Cell numbers were determined at each experimental time point after transfection using the CellTiter 96 cell proliferation assay (Promega, Madison, WI).

### Lung metastasis in nude mice

Animal experiments were approved by the Institutional Animal Care and Use Committee (IACUC) of Emory University. Five- to six-week old female athymic (nu/nu) mice were ordered from Harlan (Indianapolis, IN) and housed under pathogen-free conditions in microisolator cages with laboratory chow and water *ad libitum*. Both 801C-pLKO.1 and 801C-shDR5 cells (1 × 10^6^ cells in 100 μl PBS) were injected subcutaneously into the flank region of nude mice. Tumor volumes were measured using caliper measurements once every 3 days and calculated with the formula *V* = length × width^2^/2. When tumor volumes reached a size of approximately 3000 mm^3^, the mice were sacrificed, since our preliminary study suggested that lung metastasis is likely to be seen in mice with larger tumors. The lungs were then collected, fixed in 10% formalin, paraffin embedded, and sectioned. Three 6 μm sections (first one collected for every 10 slices) were stained with H&E to evaluate the presence of lung metastasis.

### Statistical analyses

The statistical significance of differences between two groups was analyzed with two-sided unpaired Student's *t* tests when the variances were equal or with Welch's corrected *t* test when the variances were not equal by use of Graphpad InStat 3 software (GraphPad Software, San Diego, CA). In addition, Fisher's exact test was used for analysis of contingency tables with the same software. Results were considered to be statistically significant at *P* < 0.05.

## SUPPLEMENTARY FIGURE


