# Supplementary material for: Suppression of death receptor 5 enhances cancer cell invasion and metastasis through activation of caspase-8/TRAF2-mediated signaling
Source: Oncotarget. 2015 Oct 15;6(38):41324–38. doi: 10.18632/oncotarget.5847 (PMC4747408; doi:10.18632/oncotarget.5847)
Supplement: Supplementary file 1 [file oncotarget-06-41324-s001.pdf]

SUPPLEMENTARY FIGURE

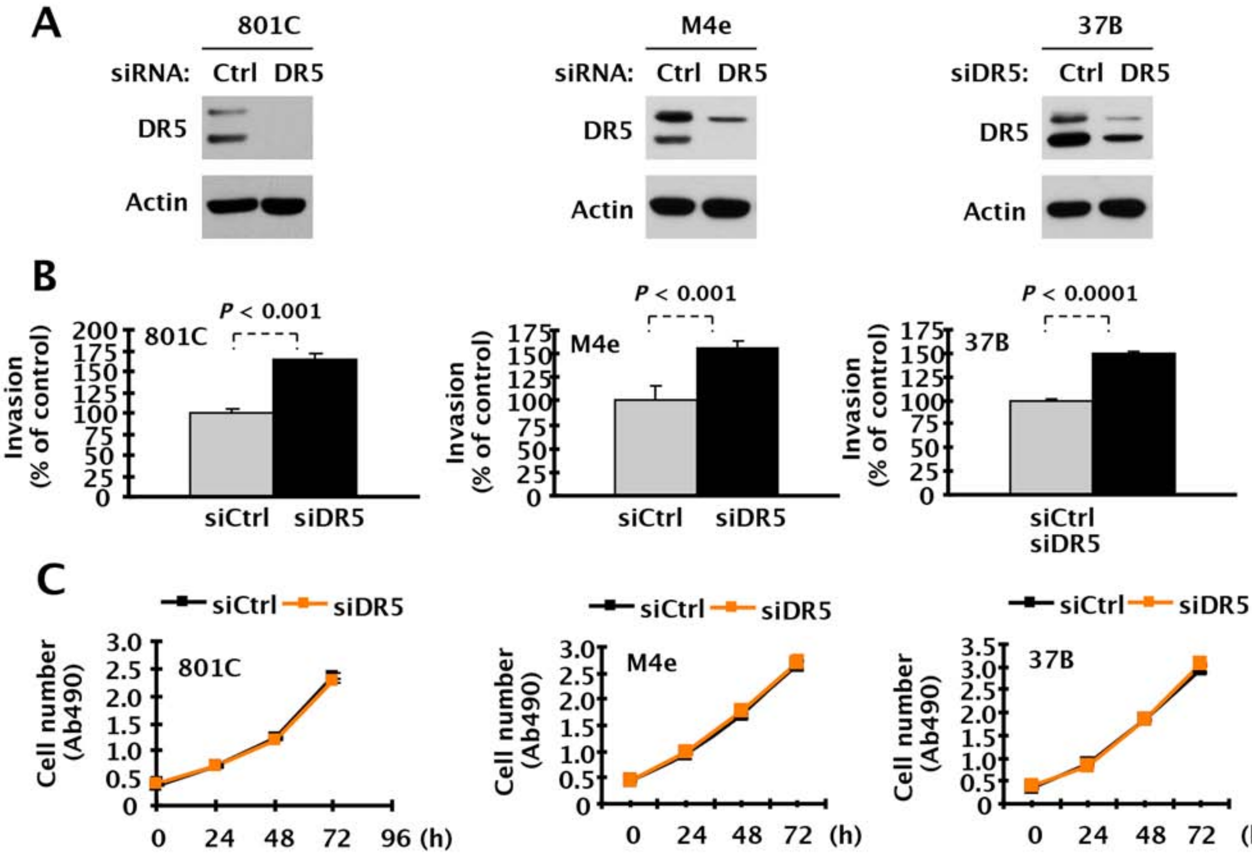

Supplementary Figure S1: DR5 knockdown increases invasion of cancer cells without affecting cell growth. 801C, M4e or 37B cells transiently transfected with control (Ctrl) or DR5 siRNA were plated in 12-well plates for evaluating DR5 expression with Western blotting **A.** in the Matrigel invasion chambers for invasion assay **B.** and in 96-well plates for cell growth measurement with the MTS assay **C.** after approximately 48 h incubation. The data are means ± SDs of triplicate determinations.
